# Effect of micronutrient iron on bioactive compounds isolated from cryptophytes

**DOI:** 10.3389/fpls.2023.1208724

**Published:** 2023-07-27

**Authors:** Maryam Abidizadegan, Jaanika Blomster, Elina Peltomaa

**Affiliations:** ^1^ Ecosystem and Environment Research Program, Faculty of Biological and Environmental Sciences, University of Helsinki, Lahti, Finland; ^2^ Ecosystem and Environment Research Program, Faculty of Biological and Environmental Sciences, University of Helsinki, Helsinki, Finland; ^3^ Department of Forest Sciences, University of Helsinki, Helsinki, Finland

**Keywords:** antioxidant activity, bioactive compounds, cryptophytes, extracellular polymeric substances, iron, phenolic compounds, phycobiliprotein

## Abstract

Iron is one of the important micronutrients affecting algal growth due to its fundamental role in the physiological processes, including photosynthetic electron transport, respiration, and nitrogen fixation. In this study, the effect of different iron levels on growth and the production of bioactive compounds (phycoerythrin (PE), extracellular polymeric substances (EPS), and phenolic compounds (PCs)) of five cryptophyte strains were investigated. Also, the antioxidant capacity of the bioactive compounds was explored. The results showed species-specific responses to the impact of iron on growth of cryptophytes and accumulation of bioactive compounds. The growth rates of *C. pyrenoidifera* and *Cryptomonas* sp. varied significantly at different iron levels, and a reduction in the PE content was observed for several cryptophytes cultured at the highest iron level. However, no significant differences were detected in EPS content at different iron levels. Differences in PC contents of *C. pyrenoidifera* and *Cryptomonas* sp. at medium iron level were statistically significant compared with the other two treatments. The results also revealed species-specific differences in antioxidant activity at different iron levels; each studied strain followed its own pattern in response to change in iron level, and each bioactive compound had a different antioxidant activity. Overall, however, PCs demonstrated higher antioxidant activity than PE and EPS. In summary, iron has an impact on growth, bioactive compound accumulation, and antioxidant activity. However, the species-specific responses to changes in iron level should not be ignored when modifying culture conditions for optimal harvest of bioactive compounds.

## 1 Introduction

The effect of macronutrients, including nitrogen and phosphorous, on microalgal growth and their bioactive compounds has been extensively researched. However, micronutrients, such as iron, also play a fundamental role in the growth of microalgae. Iron has a crucial function in algal metabolic processes, involving photosynthetic electron transport, nitrogen fixation process, and respiration (Liu et al., 2018). The function of iron in phytoplankton cells has been explained as iron directly affecting the various methanolic pathways by its catalytic role in enzymes and indirectly contributing to energy production by high energy-carrier molecules, including NADPH and ATP (Schoffman et al., 2016).

Although the effect of iron on algal growth has been established, little research has focused on the impact of iron on the accumulation of bioactive compounds. Growth rates of certain microalgal species, such as the green algae *Raphidocelis subcapitata*, *Dunaliella salina*, *Desmodesmus subspicatus*, *Parachlorella kessleri*, *Chlorella vulgaris*, and *Haematococcus pluvialis*, the cryptomonad *Cryptomonas* sp., and the dinoflagellate *Prorocentrum micans*, have been enhanced by increasing the iron concentration of the culture media (Weng et al., 2007; Liu et al., 2008; Padrova et al., 2015; Rastar et al., 2018). On the other hand, a study where *Chlorella* sp. was cultured at different iron levels demonstrated that it grew well at the lowest iron level (0.35 mg L^-1^) (Iriani et al., 2011).

It can be deduced from previous studies that the effect of iron on algal growth rate depends largely on algal species. Therefore, the investigation of the impact of iron on other algal groups is essential for identifying optimal culture conditions to produce high-quantity/quality bioactive compounds applicable in various industries. One of the main groups of microalgae is cryptophytes found in such diverse aquatic habitats as marine, freshwater, and brackish waters (Clay, 2015). The absence of a recalcitrant cell wall is one important feature of cryptophytes, making biomass processing and cell breaking simpler when extracting biomolecules. This benefit leads to the higher ratio of usable total biomass with biological active compounds. In fact, the lack of a heavy cell wall in cryptophytes enhances the possibility of harvesting biomass with high-quantity biomolecules instead of increasing only the biomass weight of the useless part (Mercier et al., 2022). Furthermore, cryptophytes produce several valuable bioactive compounds such as phycobiliproteins (PBPs), extracellular polymeric substances (EPS), and omega-3 polyunsaturated fatty acids, including eicosapentaenoic acid (EPA) and docosahexaenoic acid (DHA) (Giroldo et al., 2005; Peltomaa et al., 2018; Abidizadegan et al., 2022; Mercier et al., 2022). Nonetheless, the biotechnological potential and applications of cryptophytes have not been thoroughly investigated. Hence, in addition to exploring the bioactive compounds of cryptophytes and their potential, studying the optimum cultural conditions to derive bioactive compounds of high quantity and quality is necessary.

In this study, the effect of iron on biomolecules of cryptophytes, including PBPs, EPS, and phenolic compounds (PCs), is explored. Contrary to red algae and cyanobacteria, cryptophyte strains only contain one type of PBP, either phycocyanin or phycoerythrin (Hill and Rowan, 1989). This removes the necessity of isolating different PBPs from each other, and accordingly, makes the protein purification simpler in the production process of PBPs (Mercier et al., 2022). Additionally, due to the lower molecular weight, cryptophyte PBPs are more practical as labels in fluorescence medical diagnostics than cyanobacterial and red algal PBPs (Telford et al., 2001; Roman et al., 2002). The cryptophytes in this study contain phycoerythrin (PE), which is used to make natural colorant of food, cosmetics, drugs, fluorescent probes for immunoassays, and flow cytometry (Garcia et al., 2021; Chen et al., 2022). The EPS – a mixture of biopolymers, including polysaccharides, proteins, nucleic acids, and lipids – with physiochemical properties are applied in food and pharmacy as preservatives, gelling agents, and thickeners (Tiwari et al., 2020). EPS can also be used as an emulsion stabilizer in food and as a hydrating agent in pharmaceuticals and cosmetics (Xiao and Zheng, 2016). Moreover, permeability properties of EPS make them advantageous as plant biostimulants to promote sustainable agriculture (Rachidi et al., 2020). The PCs are considered to be natural antioxidants applicable in pharmaceutical, cosmetic, and food industries with health-promoting effects to prevent such diseases as cancer and inflammatory disorders (Cory et al., 2018). Although there are several studies on the antioxidant activity of algal PE, EPS, and PCs (Kim et al., 2018; Punampalam et al., 2018; Frazzini et al., 2022; Mousavian et al., 2022; Wang et al., 2022), cryptophyte algae have not yet been explored in terms of antioxidant activity of bioactive compounds.

Several studies have presented the effect of different iron levels on the above-mentioned bioactive compounds of certain algae. For example, the highest PBP content of the red macroalga *Gracilaria tenuistipitata* has been reported with high iron content in tissue (549 µg dw^-1^) (Liu et al., 2000). Contrary to this, cyanobacterium *Arthrospira platenis* has shown a negative correlation with iron concentration (Akbarnezhad et al., 2016). Additionally, the highest total PC content of the green alga *Chlorella* sp. after a 14-day cultivation occurred at the highest iron level (13.99 mg L^-1^), while after 21 days it was high at the lowest iron level (0.35 mg L^-1^) (Iriani et al., 2011). The aim of this investigation is to better understand the relationship between culture conditions at different iron levels and the accumulation of bioactive compounds, such as PBP, EPS, and PCs, of cryptophyte algae. Growth and biomass productivity changes of cryptophyte algae under different iron levels, and the antioxidant activity of studied bioactive compounds were explored. Finally, promising cryptophyte strains for producing high amounts of bioactive compounds were identified.

## 2 Materials and methods

### 2.1 Algae and growth conditions

Five cryptophyte strains (**Table 1**) were grown in culture media with three different iron levels. Culture media used for the marine strain *Rhodomonas salina* was F/2 and for freshwater *Cryptomonas* strains MWC (Guillard and Ryther, 1962; Guillard and Lorenzen, 1972). The iron source in both applied media was FeCl_3_ at a concentration of 3.15 mg L^-1^ (control and medium level). By manipulating the media, two other iron treatments contained 1.75 mg L^-1^ (low level) and 6.3 mg L^-1^ (high level). The changes in iron content of the algal biomass caused by the treatments were confirmed by iron analysis (see **Supplemental Table 1**). The experiment was conducted in four replicates, and in 2 L glass bottles, which were randomly arranged in growth cabinets (Friocell Evo 404, MMM Group, Germany) for ten days. Strains were kept at 20°C, under a 16 h: 8h light/dark cycle at a light level of 100 µmol photons m^-2^ s^-1^, and 2% (v/v) CO_2_ level.

**Table 1:** Cryptophyte strains, culture collection codes, and habitats of each strain.

Strain	Code in culture collection	Habitat
*Rhodomonas salina*	CCMP 757	Marine
*Cryptomonas pyrenoidifera*	CCAP 979/61	Freshwater
*Cryptomonas curvata*	CCAP 979/63	Freshwater
*Cryptomonas ozolinii*	UTEX LB 2782	Freshwater
*Cryptomonas* sp.	CPCC 336	Freshwater

### 2.2 Growth rates and biomass yield

Algal growth rate was determined by measuring optical density at 750 nm (OD_750_) five times (day 0, 1, 3, 6, and 9) during the experiment with a SHIMADZU UV-2401 PC spectrophotometer. The specific growth rate (µ_exp_; d^-1^) was estimated using Equation (1):


(1) $${µ}_{exp}=\;ln\;\left(\alpha_{f}/\alpha_{i}\right)/\left(t_{f}–\;t_{i}\right)$$

where *α_f_* and *α_i_* are the absorbance reading at the end and the beginning of the exponential growth phase, at initial time (t_i_) and final time (t_f_), respectively.

Whatman filter papers (GF/C, 47 mm diameter, ca 1.2 µm pore size) were used in dry weight determinations. The filter papers were dried at 105°C overnight and weighed. Of the cultures, 20 mL was filtered using a vacuum filter, and then the filters were dried overnight at 105°C again and reweighed.

### 2.3 Extraction and measurement of bioactive compounds

For PBPs, 40 mL of cultures were centrifuged at 4000 × g for 10 min (Heraeus Multifuge 1 S-R, Kendro Laboratory Products, Germany) to obtain pellets. The pellets were dissolved in 5 mL of 0.1 M phosphate buffer and kept at -20°C to break the cells and release the PBPs. The samples were thawed at 5°C overnight and then centrifuged at 4000 × g for 15 min to remove cell debris. Harvested supernatants were used for PBP analysis by measuring the absorption spectra. Absorbance of extracts was measured from 280 to 750 nm using spectrophotometry (Shimadzu UV-2401 PC spectrophotometer, Shimadzu Corporation, Japan) and 1 cm cuvettes against phosphate buffer as a blank sample. Finally, the PBP concentrations (µg L^-1^) were calculated using Equation (2) (Lawrenz et al., 2011; Cunningham et al., 2019):


(2) $$c=\frac{A}{\varepsilon d}\times MW\times \frac{V_{buffer}}{V_{sample}}\times 10^{6}$$

where A = subtracting the absorbance at 750 nm from the absorbance maximum of the phycoerythrin peak (548 nm for marine strain and 565 nm for freshwater strains), ε = the molar extinction coefficient for cryptophyceae PE (5.67 × 10^5^ L mol^-1^cm^-1^), MW = the molecular weight of cryptophyceae PE (45000 g mol^-1^), and V_buffer_ and V_sample_ = the volume of the buffer and sample, respectively.

For EPS, freeze-dried biomass was dissolved in 5 mL of deionized water and shaken for 20 min. After centrifuging of samples at 4000 × g for 15 min, pellets were mixed in 5 mL of 0.05% NaCl solution and placed in an overhead shaker at 60°C for one hour. After sonication of samples for 10 min at 100 W and 20°C, the suspensions were centrifuged for 15 min at 4000 × g. Finally, supernatants were lyophilized for 48 h at -60°C and 0.6 mbar and the weight of EPS was determined gravimetrically (Chang et al., 2019; Strieth et al., 2020).

For PCs, the mixture of dry biomass and methanol as solvent was sonicated at 50 Hz and 37°C for 15 min. Samples were incubated at room temperature and after one hour were centrifuged at 4000 × g for 15 min (Irondi et al., 2012; Korzeniowska et al., 2020). The Folin-Ciocalteu (FC) procedure was used to measure PCs. Folin-Ciocalteu reagent (1.5 mL) and 7.5% Na_2_CO_3_ solution (1.2 mL) were added to the sample extract (300 µL). After incubation of samples in the dark for 30 min, the absorbance was read at 765 nm. PC contents (C) were measured in milligrams gallic acid equivalent (GAE) per gram of algae dry weight using the calibration curve in mg ml^-1^ (Y= 0.014 x + 0.15, R^2^ = 0.9898) (Irondi et al., 2012; Korzeniowska et al., 2020).

### 2.4 Antioxidant capacity of bioactive compounds

To determine antioxidant capacity, DPPH (2,2-diphenyl-1-picrylhydrazyl) solution (0.1mM) was used. Extracts of bioactive compounds (0.5 mL) were mixed with 3 mL of DPPH solution. After incubating the mixture in the dark for 30 min, the absorbance (A) was read at 517 nm. The results were expressed as antioxidant capacity equivalent ascorbic acid (AEAA) using Equation (3) (Leong and Shui, 2002):


(3) $$\begin{array}{c} AEAA\;\left(mg\;AA\;g^{-1}bioactive\;compounds\right)=\left(\left(A_{control}–A_{sample})/(A_{control}–A_{AA}\right)\right)\\ \times conc.\;AA\left(mg\;mL^{-1}\right)\times vol\;extract\;\left(mL\right)/used\;bioactive\;compounds\;\left(g\right) \end{array}$$

where A_control_ is the absorbance of the methanolic DPPH solution in the presence of the extract.

### 2.5 Statistical analyses

The differences between the growth rates, biomass productivity, and bioactive compounds content between the studied cryptophytes were analyzed with ANOVA and Tukey *post-hoc* test’s honestly significant difference (HSD) *post hoc* test. The significance level was set to *p*< 0.05. All statistical analyses were performed using IBM SPSS 26 Statistical package (SPSS Inc., Chicago, IL, USA).

## 3 Results

### 3.1 Specific growth and biomass productivity

**Figure 1:**
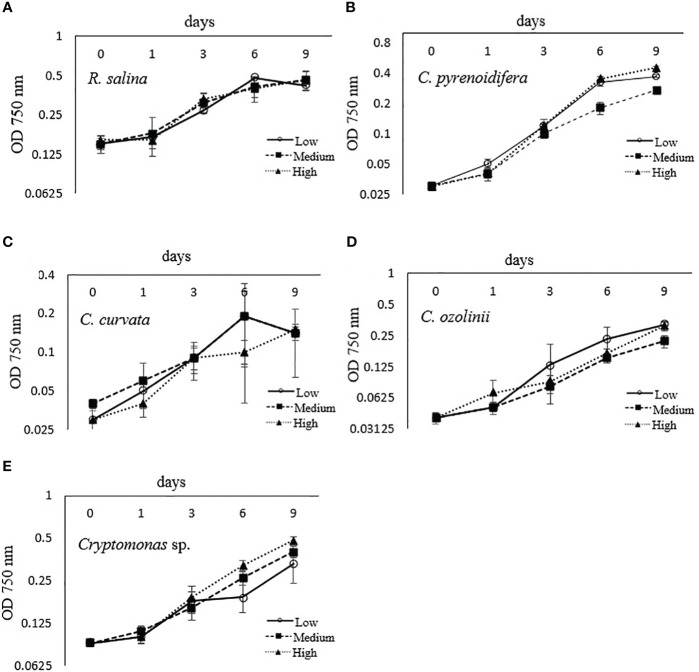
Growth curve of studied cryptophyte strains [**(A)** *R. salina*, **(B)** *C. pyrenoidifera*, **(C)** *C.* 685 *curvata*, **(D)** *C. ozolinii*, **(E)** *Cryptomonas* sp.] at different iron levels (Low:
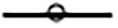
, Medium:
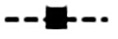
, High:
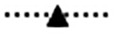
) measured at 750 nm.

**Figure 2:**
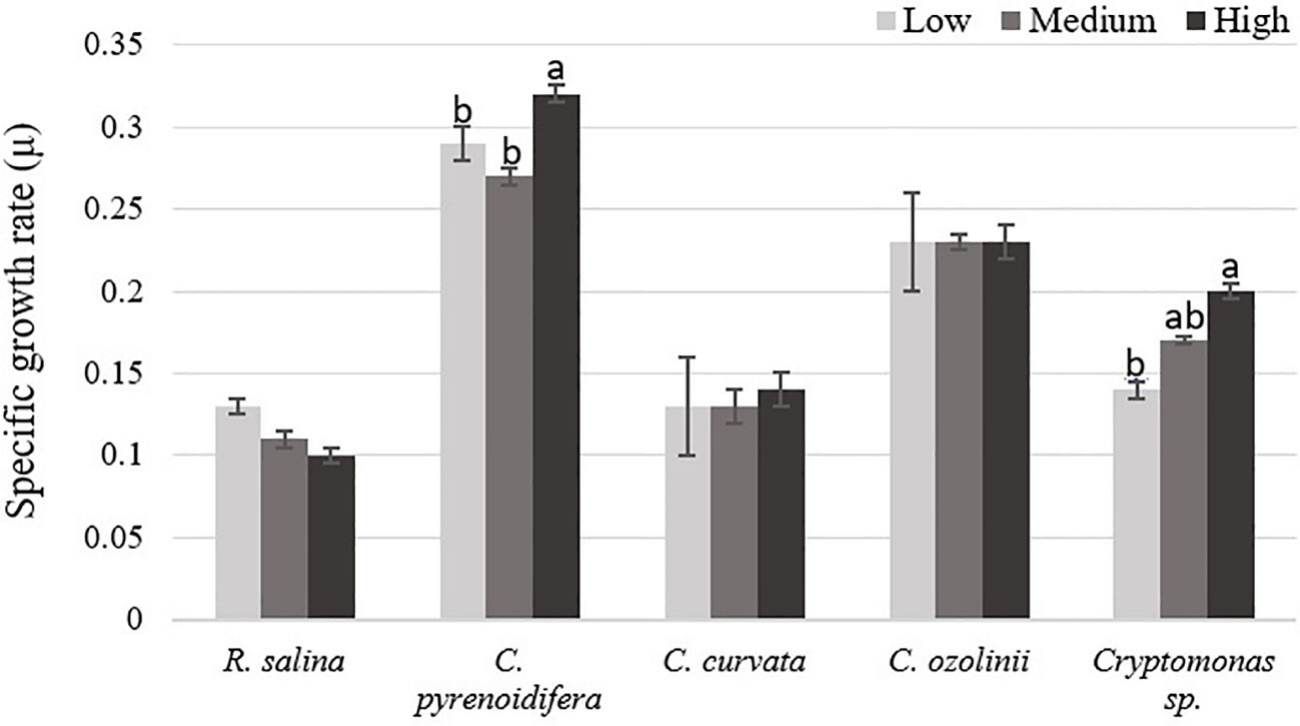
Specific growth rate (µ) of studied cryptophytes species at different iron levels. Significant differences between samples are indicated with different letters as determined by ANOVA comparison (p**<** 0.05).

Each studied strain had a specific growth pattern at different iron levels. *C. pyrenoidifera*, *C. ozolinii*, and *Cryptomonas* sp. continued to grow steadily over the experiment. However, the growth of *R. salina* at low iron level and *C. curvata* at low and control (medium) iron levels decreased after day 6 (**Figures 1A–E**). In *C. pyrenoidifera* and *Cryptomonas* sp., a significant difference emerged between the growth rates at different iron levels: the highest growth rate of these two cryptophyte strains was at high iron level (*p* > 0.05). Overall, the highest and lowest growth rates were observed in freshwater cryptophyte *C. pyrenoidifera* (~0.3 day^-1^) and marine cryptophyte *R. salina* (~0.1 day^-1^), respectively (**Figure 2**).

**Figure 3:**
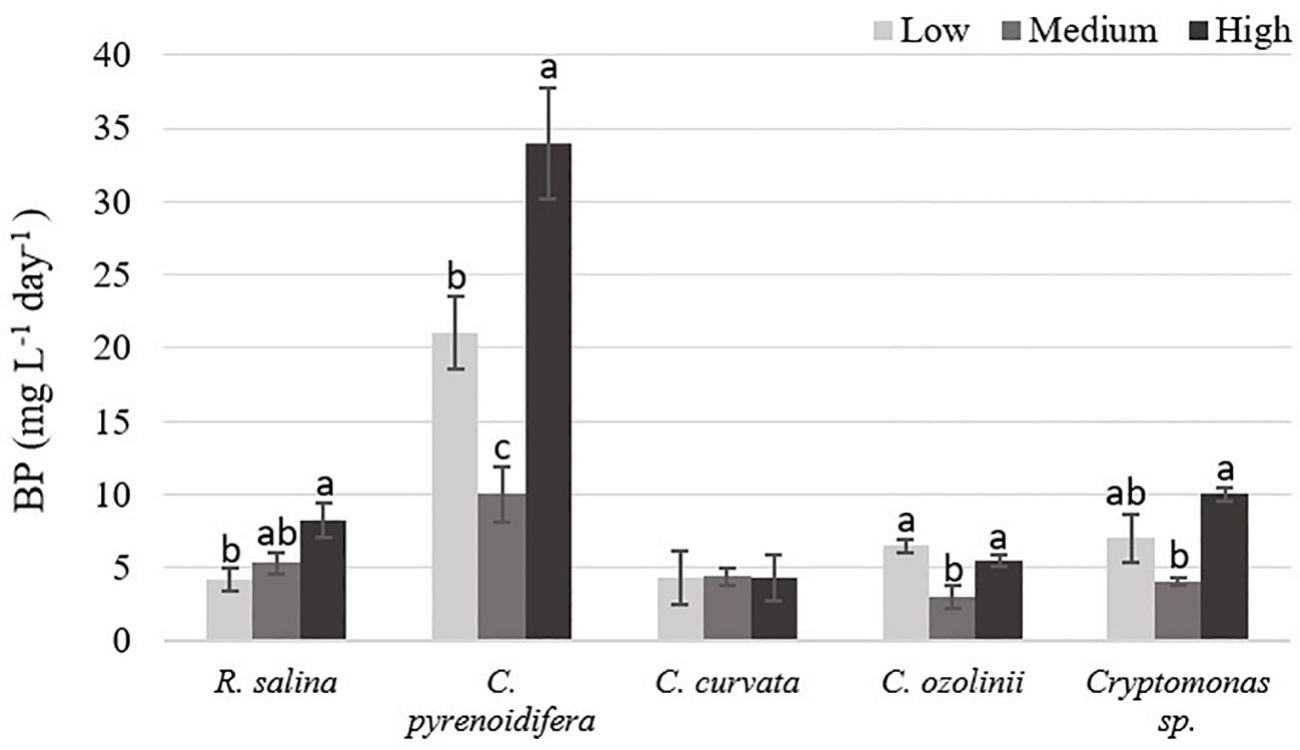
Biomass productivity (BP: mg L^-1^ day^-1^) of studied cryptophytes species at different iron levels. Significant differences between samples are indicated with different letters as determined by ANOVA comparison (p**<** 0.05).

There was a significant difference between biomass productivity of the studied cryptophytes cultured at different iron levels (*p*< 0.05), except *C. curvata*. The highest biomass production was at high iron level for most of the strains. Biomass production of *R. salina* increased gradually with rising iron levels, as biomass productivity at high iron level was ~ 55% and 95% higher than at control and low iron levels, respectively. However, biomass production of *C. pyrenoidifera*, *C. ozolinii*, and *Cryptomonas* sp. decreased at control (medium) iron level and was lower than at low or high iron level treatments. Biomass productivity of *C. pyrenoidifera* at low and high iron levels was ~110% and 240% higher than at control iron level, respectively. Regarding to *C. ozolinii*, biomass productivity at low and high iron levels was ~116% and 83% higher than at control iron level, respectively. Although the highest biomass yield of *C. ozolinii* was at low iron level, it made no significant difference to the biomass yield of *C. ozolinii* at high iron level (*p*< 0.05; **Figure 3**). Additionally, *Cryptomonas* sp. demonstrated that biomass productivity at low and high iron levels was ~75% and 150% higher than at control iron level, respectively.

### 3.2 Bioactive compounds

#### 3.2.1 Phycobiliprotein

**Figure 4:**
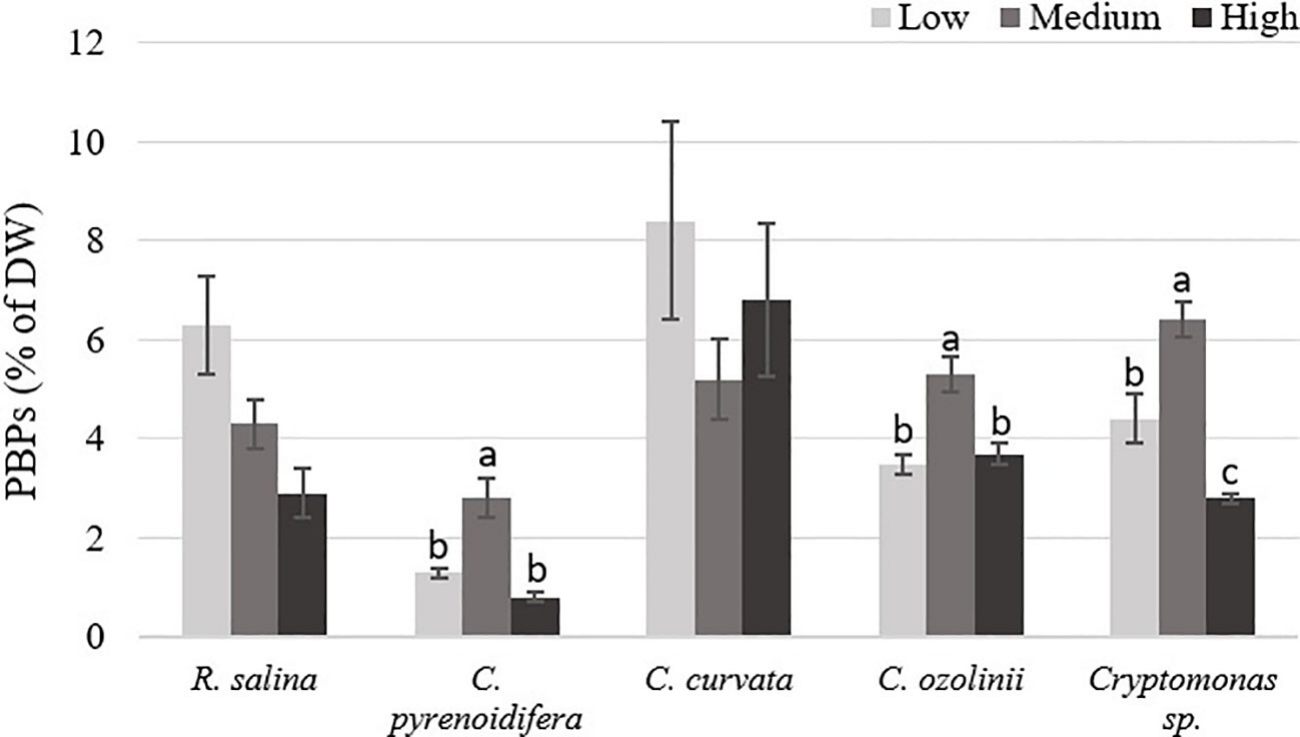
Phycobiliproteins (PBPs) content of studied cryptophytes species expressed as percentage of dry weight at different iron levels. Significant differences between samples are indicated with different letters as determined by ANOVA comparison (p< 0.05).

Phycobiliprotein content isolated from each strain grown at different iron levels showed a significant difference (*p*< 0.05), excluding *R. salina* and *C. curvata*. The highest PE content of *R. salina* and *C. curvata* was at low iron level, but without significant difference compared with other treatments (*p* > 0.05). However, for *C. pyrenoidifera*, *C. ozolinii*, and *Cryptomonas* sp. the medium iron level was the most favorable condition for PE production (**Figure 4**).

**Figure 5:**
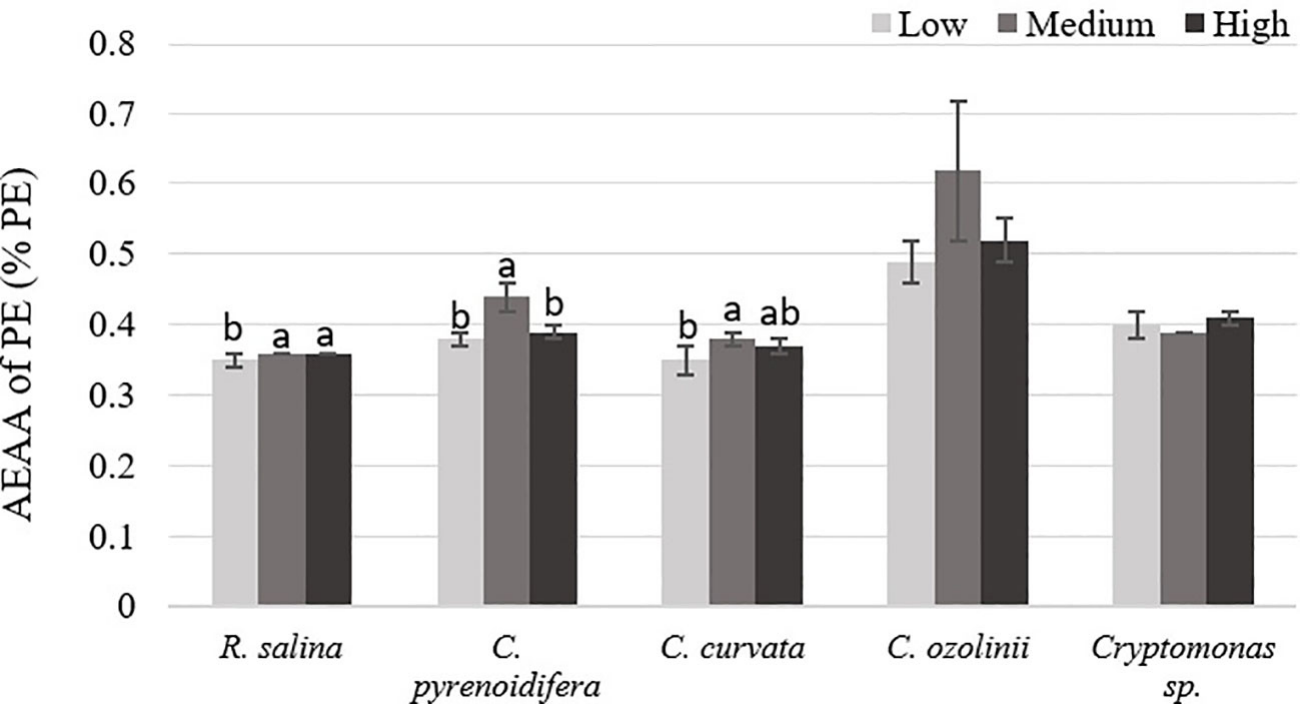
Antioxidant capacity (AEAA) of PE (% PE) derived from the studied cryptophytes cultured at different iron levels. Significant differences between samples are indicated with different letters as determined by ANOVA comparison (p**<** 0.05).

Antioxidant capacity of PE had significant differences at different iron levels in *R. salina*, *C. pyrenoidifera*, and *C. curvata* (*p*< 0.05). There was a significant difference between antioxidant activities of PE at medium iron level with two other iron levels in *C. pyrenoidifera*. Additionally, in *R. salina* and *C. curvata*, a difference was observed between medium and low iron levels (*p*< 0.05). The highest antioxidant capacity of PE was at medium iron level for all studied cryptophyte strains, except *Cryptomonas* sp. (**Figure 5**). The PE of *C. ozolinii* contained the highest antioxidant capacity among the studied cryptophytes (*p*< 0.05).

#### 3.2.2 Extracellular polymeric substances

**Figure 6:**
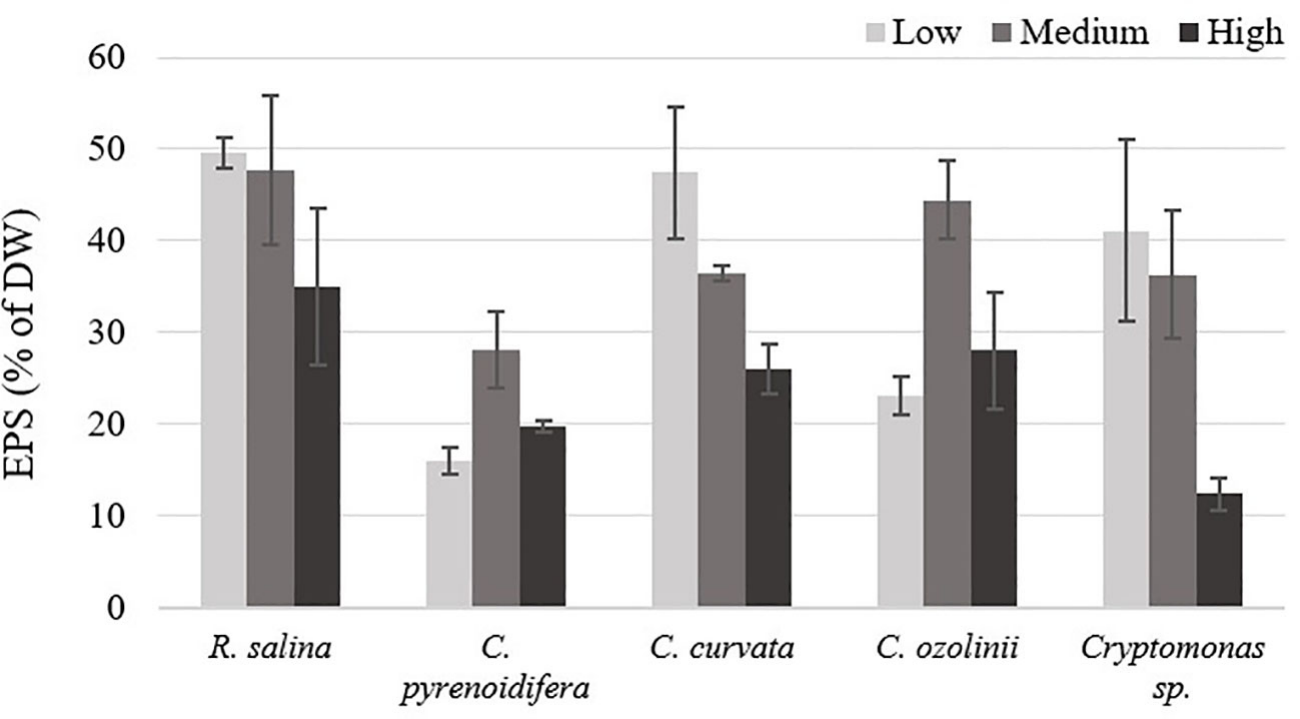
Extracellular polymeric substances (EPS) content of studied cryptophytes species expressed as percentage of dry weight at different iron levels.

There was no significant difference among EPS isolated at different iron levels for the studied strains (p > 0.05; **Figure 6**). Whereas *R. salina*, *C. pyrenoidifera*, and *Cryptomonas* sp. produced the highest EPS content at low iron level, the highest quantity of derived EPS for *C. pyrenoidifera* and *C. ozolinii* was at control iron level. Among the studied cryptophyte strains, *R. salina* produced the highest EPS with ~50% dry biomass.

**Figure 7:**
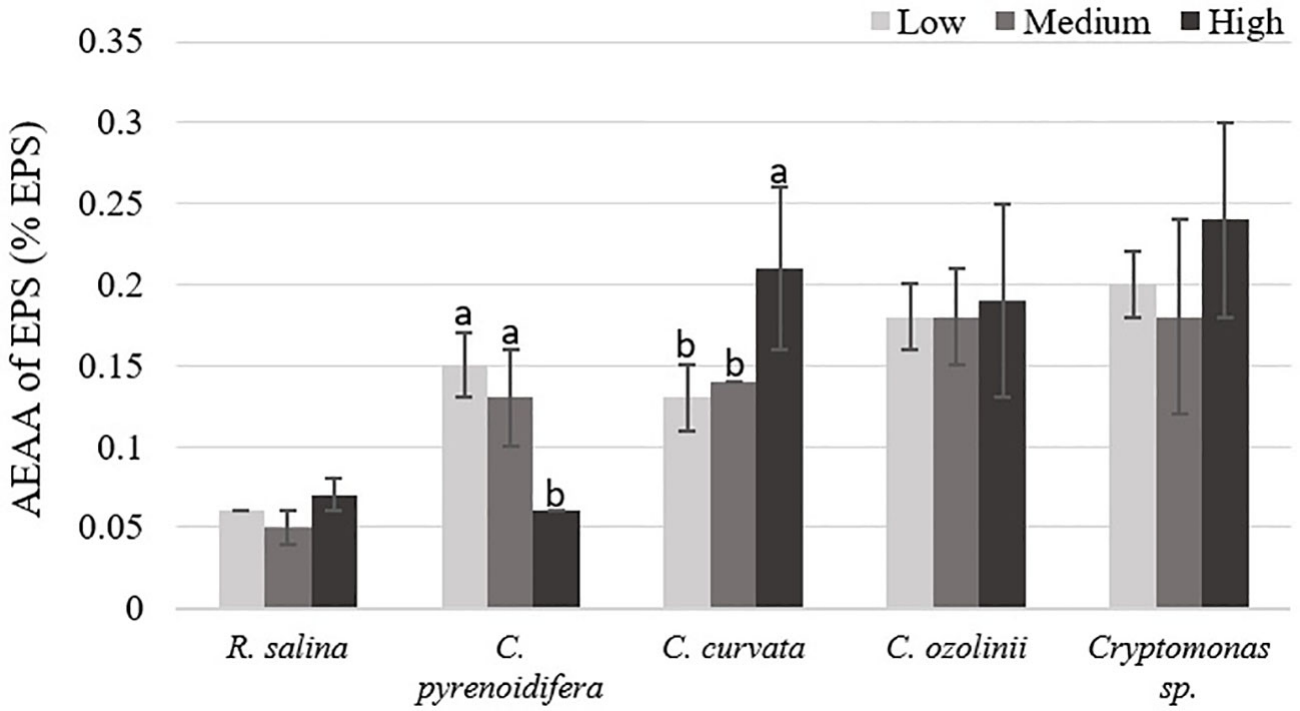
Antioxidant capacity (AEAA) of EPS (% EPS) derived from the studied cryptophytes cultured at different iron levels. Significant differences between samples are indicated with different letters as determined by ANOVA comparison (p< 0.05).

In *C. pyrenoidifera* and *C. curvata*, antioxidant activity of EPS was significantly different between the high iron level and the other two iron levels (p< 0.05). Although the highest antioxidant activity of EPS for *C. pyrenoidifera* was at low iron level, *C. curvata* grown under high iron level showed the highest antioxidant activity of EPS. However, there was no significant difference between antioxidant activity of EPS for *R. salina, C. ozolinii*, and *Cryptomonas* sp. cultured at different iron levels (p > 0.05; **Figure 7**). The highest and lowest antioxidant activity of EPS was observed in *Cryptomonas* sp. and *R. salina*, respectively.

#### 3.2.3 Phenolic compounds

**Figure 8:**
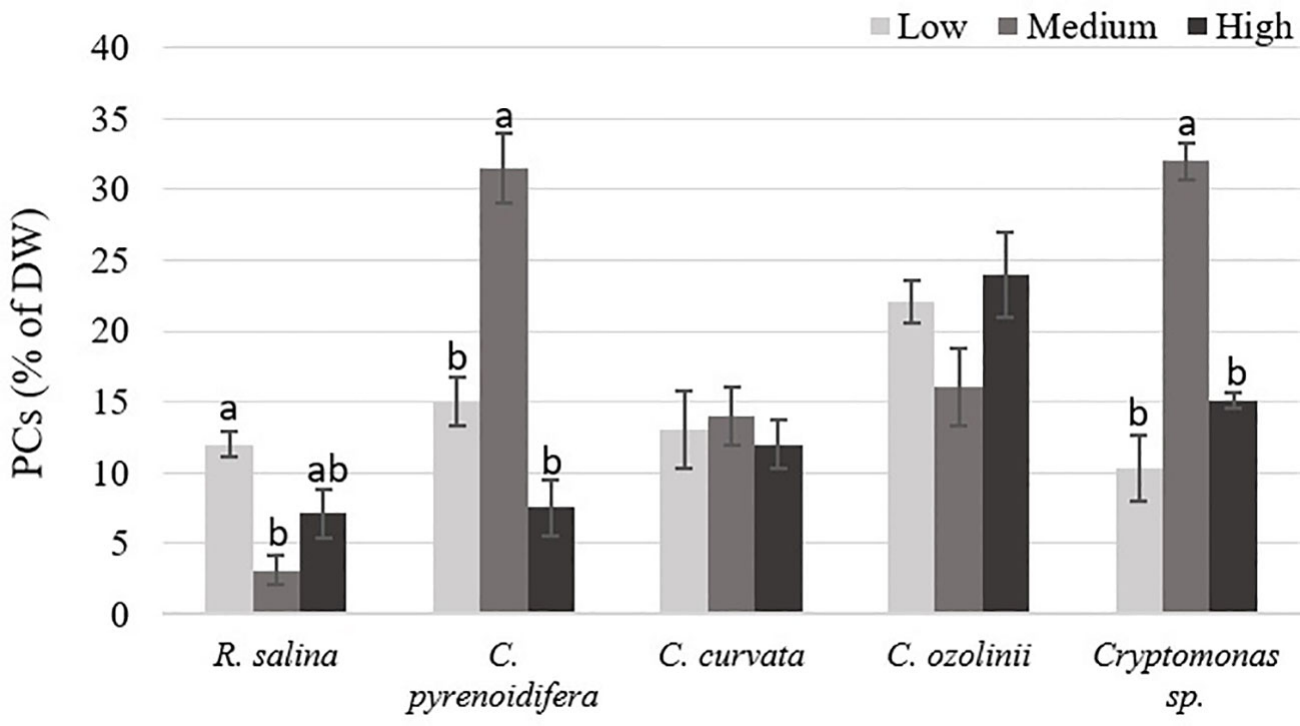
phenolic compounds (PCs) content of studied cryptophytes species expressed as percentage of dry weight at different iron levels. Significant differences between samples are indicated with different letters as determined by ANOVA comparison (p< 0.05).

Phenolic compound contents differed significantly at different iron levels in *R. salina*, *C. pyrenoidifera*, and *Cryptomonas* sp. (*p*< 0.05; **Figure 8**). The highest PC content of *R. salina* and *C. ozolinii* was at low and high iron levels, respectively, whereas *C. pyrenoidifera*, *C. curvata*, and *Cryptomonas* sp. produced the highest PC content at medium iron level (**Figure 8**). Overall, among the studied strains, *C. pyrenoidifera* and *C. ozolinii* grown at medium iron level produced the highest quantity of PCs with almost 32% PCs of dry biomass (**Figure 8**).

**Figure 9:**
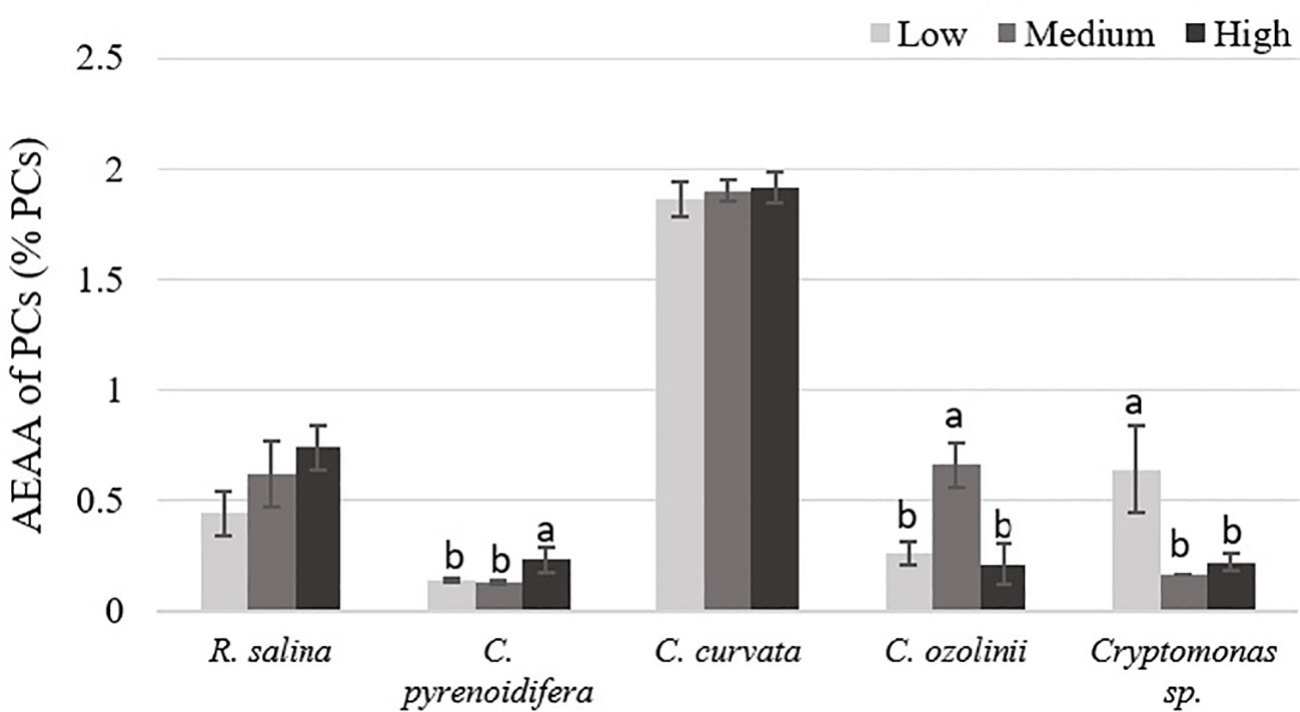
Antioxidant capacity (AEAA) of PCs (% PCs) derived from the studied cryptophytes cultured at different iron levels. Significant differences between samples are indicated with different letters as determined by ANOVA comparison (p< 0.05).

Except for *R. salina* and *C. curvata*, other studied cryptophytes showed significant differences in antioxidant activities of PCs at different iron levels (*p*< 0.05). Although *Cryptomonas* sp. contained the highest antioxidant capacity of PCs at low iron level, PCs extracted from *R. salina* and *C. pyrenoidifera* had the maximum antioxidant activity at high iron level. Moreover, *C. ozolinii* showed the highest antioxidant activity of PCs at medium iron level (**Figure 9**). The PCs of *C. curvata* contained the highest antioxidant capacity among the studied cryptophytes (*p*< 0.05).

## 4 Discussion

Here, we studied the relationship between culture conditions at different iron levels and growth as well as accumulation of bioactive compounds such as PBP, EPS, and PCs of cryptophyte algae. A significant difference emerged in growth rate of *C. pyrenoidifera* and *Cryptomonas* sp. at different iron levels. Similarly, some other studies have shown a higher growth rate with increased iron levels, e.g. the green alga *C. vulgaris* and flagellate *Gonyostomum semen* cultures grew better at high iron levels (Liu et al., 2008; Munzner et al., 2021). By contrast, the eustigmatophyte *Nannochloropsis salina*, haptophyte *Pleurochrysis carterae*, and *Chlamydomonas* sp. showed their highest growth rate at low iron levels (Tiamiyu, 2011; Munzner et al., 2021). Furthermore, Weng et al., 2008 found that iron deprivation could lead to slower growth rate in *Cryptomonas* sp. because iron deficiency causes misalignment of chloroplast lamellae, metamorphosis, and twisted thylakoids. It may also weaken algal photosynthesis and general metabolism.

Nevertheless, our practical outcomes indicate that the change in iron level did not have a noticeable impact on the growth rate of the studied cryptophytes, particularly *R. salina*, *C. curvata*, and *C. ozolinii*. Thus, the lowest iron level in our study appears adequate to fulfill the iron requirements for growth of cryptophytes. Consistent with this, Isani et al., 2022 reported no significant difference in growth of *Arthrospira platensis* cultivated at different iron levels (0.84, 3.44, and 6.90 mg L^-1^). In addition, levels of iron ranging from 1.9 to 10 mg L^-1^ showed no significant effect on the growth of *A. platensis* (Ismaiel et al., 2018). Iron uptake by microalgae depends on the iron demand of the cell and the iron uptake rate, both of which are controlled by cell size. Species with smaller cell size achieve faster iron uptake rate per cell volume than those with larger cells and are thus better at fulfilling the iron demand for growth (Marchetti and Maldonado, 2016). Our results support these findings, showing that iron uptake and consequently the growth rate and biomass productivity of *R. salina* with an extremely small cell size [5 µm, with 0.02% dry weight iron content (**Supplemental Table 1**)] are similar to *C. curvata* with a large cell size (up to 50 µm, with 0.01% dry weight iron content) under low iron level. Moreover, *C. pyrenoidifera* [15−25 × 10−13.5 × 9−12 µm, 0.01% dry weight iron content (**Supplemental Table 1**)] and *C. ozolinii* [17−29 × 9−13 × 6−9 µm, 0.03% dry weight iron content (**Supplemental Table 1**)] with smaller cell size than *C. curvata* (35−44 × 18−20 × 17−19 µm) have a higher growth rate and biomass productivity specifically under low iron level. Overall, species with small cell size can cope better with low iron concentration than species with large size (Marchetti and Maldonado, 2016).

In terms of biomass productivity, high iron level resulted in higher biomass productivity in *R. salina*, *C. pyrenoidifera*, and *Cryptomonas* sp. Previous studies on *Chlorella vulgaris* and *Scenedesmus obliquus* demonstrated an increase in biomass production at high iron level (Liu et al., 2008; Abd El Baky et al., 2012). Marine species *R. salina* shows an increasing trend in biomass production with increased iron concentration, while biomass production of *C. curvata* is unaffected by changes in iron level. Hence, the physiological differences of species influence the response to changes of iron concentration. Another reason for lower biomass in iron deficiency is related to the biochemical processes of iron. At the level of iron deficiency, iron ions are released from the functional enzyme aconitase. However, this enzyme cannot catalyze citric acid into isocitric acid, which is an essential chemical reaction in tricarboxylic acid cycle to transform saccharide into ATP (Berg and Tymo, 2002).

An increase in iron levels led to the reduction of phycobiliprotein in some of the studied strains. The cultures of *R. salina* showed that by increasing the iron level, the accumulation of phycobiliproteins decreased. This is similar to the results of a study on the cyanobacterium *Arthrospira platensis* (Akbarnezhad et al., 2016). Moreover, phycobiliprotein content at control level was higher than at low and high iron level for *C. pyrenoidifera*, *C. ovata*, and *C. curvata*. Iron is a fundamental micronutrient in phycobiliprotein pigment biosynthesis and a component of the photosynthetic system (Geider and La Roche, 1994). Therefore, its concentration can directly affect the accumulation of phycobiliproteins in microalgae. On the other hand, the location of phycobiliproteins has a significant impact on the absorption of trace elements, particularly iron. In red algae, phycobiliproteins are placed in phycobilisomes, which are anchored to thylakoid membranes (from the stromal or cytosolic side), and phycoerythrin is located in the peripheral areas of the phycobilisomes (Talarico, 1996; Gould et al., 2007). However, in cryptophytes, due to the lack of phycobilisomes and a cell wall, phycobiliproteins are situated in lumenal side of thylakoid membrane, and consequently, their sensitivity to trace elements, including iron, is increased (Kiran and Thanasekaran, 2011; Green, 2019; Spangler et al., 2022).

Although no significant difference existed among EPS at different iron levels due to high deviations between the replicates, visual inspection of the results suggests that high iron level could have a negative effect on EPS accumulation. No reports are available on the effects of iron on EPS production. However, there are a few reports of the effect of iron on its constituent compounds containing protein, lipid, and carbohydrates. Increasing the iron concentration led to lipid accumulation in the green alga *Chlorella vulgaris* (Liu et al., 2008), suggesting that iron can have an effect on enhancing the lipid content in microalgae (Rana and Prajapati, 2021). Interestingly, the green alga *Chlamydomonas reinhardtii* produced the highest lipid content under severe iron deficiency (Devadasu and Subramanyam, 2021). In addition, iron had a negative impact on protein and carbohydrate accumulation, as low iron concentration resulted in high protein content in *Chlorella* sp. and high carbohydrate production in *C. reinhardtii* (Iriani et al., 2011; Devadasu and Subramanyam, 2021). However, a study of the effect of iron on biochemical content derived from a mix culture of microalgae revealed a positive correlation between iron concentration and protein content (Ermis et al., 2020). These different reactions of constituent compounds of EPS to changes in iron concentration make it difficult to understand EPS variation with iron changes in cultures.

The changes of PC content at different iron levels can arise from iron stress and this critical condition can be variable for each species. While the highest PC content of *C. pyrenoidifera*, *C. curvata*, and *Cryptomonas* sp. was at medium iron level (iron stress), *R. salina* and *C. ozolinii* produced the highest PC content at low and high iron levels (iron stress), respectively.

The study by Lopez et al., 2015 showed that by increasing iron level the quantity of total PCs of *Dunaliella tertiolecta* diminished. When the alga was under iron stress, the growth and production of biomass decreased, while the quantity of polyphenols increased (Lopez et al., 2015). This is consistent with our results for *R. salina*, *C. pyrenoidifera*, and *Cryptomonas* sp. On the other hand, the PC content of the diatom *Phaeodactylum tricornutum* under high iron level has been shown to be higher (Rico et al., 2012), similar to our study of *C. ozolinii*. This can be due to the role of PCs in controlling osmotic stress. Under osmotic stress, by binding polyphenols to metal ion, polyphenols act as chelate towards metals, protecting cells from metal toxicity and oxidative damage (Jung et al., 2003). According to Isani et al., 2022, there was no significant difference in PC content of *A. platensis* at different iron levels, similar to our results for *C. ozolinii* and *C. curvata*.

Overall, the quantity of PCs and iron concentration in the cell have a negative relationship (Lopez et al., 2015). A high concentration of PCs prevents excessive entrance of iron to cells. The effect of PCs as organic ligands to form a complex with Fe^3+^ and to reduce Fe^3+^ to bioavailable Fe^2+^ has been proven (Rose and Waite, 2003). Thus, at low Fe^2+^ concentration the biomass production declines steeply (Khamoushi et al., 2020). Outcomes of this study for *R. salina*, *C. pyrenoidifera*, and *Cryptomonas* sp. confirm this connection. Although there is limited literature on the effect of different iron concentrations on PC content of microalgae, especially cryptophytes, based on physiological characteristics of each species, they can have a distinct response to iron changes in cultures. Besides the impact of iron levels on PC production, physiological characteristics of each species can be effective in producing PCs under different iron concentrations. Excessively low or high concentrations of iron induce stress, which can inhibit the metabolism of the species (Xing et al., 2007). However, the limits for excessively low and high iron concentrations are different for each species and could have a distinct effect on growth and biochemical accumulation in each species.

According to Iriani et al., 2011, in addition to iron level, culturing time can affect PC production. In the study by Iriani et al., 2011, on *Chlorella* sp. after 7 days of incubation under different iron levels (0.35, 4.89, 9.44, and 13.99 mg L^-1^), the highest PC content was at 0.35, followed by 9.44 mg L^-1^ iron concentrations, consistent with our results not showing a direct correlation between iron level and PC content. However, after 14 days, the highest PC content of *Chlorella* sp. was at high iron level (13.99 mg L^-1^), and after 21 days, the highest PC content was at a lower iron level (0.35 mg L^-1^; Iriani et al., 2011). In summary, variation in culturing time can have an effect on PC production. However, this correlation has not been explored in the present study.

The results of this research indicate that the antioxidant activity of studied bioactive compounds depends on two factors. First, the antioxidant activity of each biochemical is different. Overall, PCs have higher antioxidant activity than PE and particularly EPS. Second, there are also species-specific differences; no regular pattern exists for changing the antioxidant activity at different iron levels, and each studied strain follows its own specific pattern under different culture conditions. Other studies have found that different strains of the same taxa show different antioxidant capacity; e.g. *Cryptomonas pyrenoidifera* ACOI 1847 contains higher antioxidant capacity than *C. pyrenoidifera* ACOI 1850 (Assuncao et al., 2017).

Few reports have examined the effect of iron on the antioxidant activity of microalgae and their biomolecules. However, iron plays a key role in the production of reactive oxygen species (ROS) *via* the Harber-Weiss mechanism, the Fenton reaction, and the electron transport chain (Gauthier et al., 2020). High iron concentration induces oxidative stress, and accordingly, microalga produce antioxidants as a defensive mechanism (Gauthier et al., 2020). The results of their study are confirmed by our findings of antioxidant activity of bioactive compounds. By contrast, the biomass of *Arthrospira platensis* cultivated at the lowest iron level was noted in another study to contain the highest antioxidant activity (Spangler et al., 2022). However, in their study, the antioxidant activity of the whole biomass was reported, not the antioxidant activity of specific biochemicals, which have been demonstrated as in our study.

Based on our previous study, the antioxidant activities of the investigated compounds were high (IC50< 50 µl mL-1) (Abidizadegan et al., 2022). In the present study, we also show that the quantity of antioxidants is considerable; PE derived from *C. ozolinii* is capable of producing ~6 mg EAA g^-1^ PE, EPS of Cryptomonas sp. can produce ~2.5 mg EAA g^-1^ EPS, and high antioxidant quantity is observed in PCs extracted from *C. curvata*, with ~19 mg EAA g^-1^ PCs. The results obtained for antioxidant capacity in this study are difficult to compare with those of other studies due to differences in methods and representation of data, e.g. antioxidant capacity can be presented as Trolox equivalent antioxidant capacity, ferric-reducing antioxidant power, or IC50. Additionally, unlike our study showing antioxidant activity of different algal compounds, other published studies have only evaluated the total antioxidant activity. The highest total antioxidant activities measured in microalgae are for the marine diatom *Navicula clavata* and the green microalgae *Chlorella marina* and *Dunaliella salina*, which have shown total antioxidant activity of ~0.6, 1.0, and 0.9 mg EAA g^-1^ dry weight, respectively (Hemalatha et al., 2013). A study by Haoujar et al., 2022 showed total antioxidant capacity of ~0.40 mg EAA g^-1^ dry weight for the green microalgae *Nannochloropsis gaditana* and *Tetraselmis suecica* and for the marine diatom *Phaeodactylum tricornutum*. Therefore, since antioxidant activity of PE, EPS, and PCs derived from cryptophyte algae combined is more than the total antioxidant activity of other studied microalgal species, cryptophyte algae could be a potential source of natural antioxidants for the food, cosmetic, and nutraceutical industries.

### 4.1 Commercial potential

For thousands of years, humans have been benefitting from microalgae (Koyande et al., 2019), and over the last five decades the field of microalgal biotechnology has grown and expanded significantly. However, despite recent advancements, the range of algal products available for commercial use is still relatively limited. Although progress has been made in applying microalgae to produce a wide range of bioproducts, only a small number of the vast species of microalgae has been examined. Cryptophytes are one of the promising microalgae with high nutritional value, including a rich polyunsaturated fatty acid (PUFA) profile, high protein content, and antioxidant pigments, warranting more attention in academic and industry research.

According to the results of the present study and our previous report (Abidizadegan et al., 2022), cryptophyte species are able to produce PBP pigment content of about 1-35% dry weight. Additionally, a study by Latsos et al., 2021 showed that *Rhodomonas* sp. can produce phycoerythrin content ~28% of dry weight. In comparison to well-known phycoerythrin producing strains, including *Spirulina* sp. (~1.4% DW), *Oscillatoria* sp. (1.8% DW), and *Porphyridium purpureum* (12.58% DW), cryptophytes have capability to produce higher phycoerythrin content (Ji et al., 2023). Compared with other PBP producers, including cyanobacteria and red algae, cryptophytes produce only one type of PBP by each strain, simplifying the production process of PBP. Additionally, cryptophytes possess PBP with lower molecular weight, making them practical in terms of fluorescent labeling application. The lack of a strong cell wall in cryptophytes is another advantage of this algal group to facilitate biomass processing and PBP extraction. Thus, cryptophytes could be considered a reliable source of PBP to be applied as natural color in food and cosmetic industries and as fluorescent probes and analytical reagents in biomedical science (Garcia et al., 2021; Mercier et al., 2022). Furthermore, phycoerythrin has shown pharmacological and biological properties including anti-inflammation and anti-tumor effects, as well as preventing Alzheimer disease and liver cancer (Ji et al., 2023).

High microalgal EPS productivity is essential for cost-effective global EPS production. Identifying new microalgae with high EPS yield is a priority to facilitate its industrial development. The EPS-producing capability of cryptophytes in this study is remarkable, as the amount of EPS secreted from R. salina is about 50% dry weight. Therefore, compared with the microalgal species studied to date, cryptophytes would be a rich source of beneficial EPS in industries associated with the production of paper, paint, food, textile, and drugs, among others, due to having thickening, stabilizing, and gelling capacities (Tiwari et al., 2020).

Phenolic compounds are promising substances with health-giving properties that are valuable to many industries. Cryptophyte algae produce PC content of about 30% dry weight (*C. pyrenoidifera* and *Cryptomonas* sp.) and are capable of being additional or alternative natural sources of PCs in food, pharmaceutical industries, and healthcare products (Cory et al., 2018).

Data derived from this study demonstrate that cryptophytes could be considered a natural antioxidant source. For example, phycoerythrin derived from *C. ozolinii* contains ~0.6% antioxidant capacity. The highest antioxidant content of EPS is in *Cryptomonas* sp., at ~0.2%. Phenolic compounds of cryptophytes indicate the highest antioxidant capacity, which PCs extracted from *C. curvata* involves ~2% antioxidant capacity.

## 5 Conclusion

This study investigated the effect of iron on bioactive compound accumulation and antioxidant activity of these bioactive compounds isolated from different cryptophyte strains. In addition to iron impact, each strain has a specific response to change in iron level. Based on our results, cryptophytes could be a natural source of phenolic compounds and particularly extracellular polysaccharides, in addition to the phycoerythrin pigment. The antioxidant capacity of the investigated cryptophytes was also significant. The future of cryptophytes thus looks bright and their potential in various applications should be considered. Improving the growth rate of cryptophytes will be a challenge, but it can be addressed through a better understanding of their physiology and optimizing the culture conditions and procedures, including photobioreactors.

## Data availability statement

The raw data supporting the conclusions of this article will be made available by the authors, to any qualified researcher with reasonable request.

## Author contributions

MA performed the research, analyzed the data, and wrote the manuscript. EP and JB contributed to the study design and manuscript revisions. All authors have reviewed, discussed and agreed to the authorship and submission of the manuscript.
