# Supplementary material for: Effect of micronutrient iron on bioactive compounds isolated from cryptophytes
Source: Front Plant Sci. 2023 Jul 27;14:1208724. doi: 10.3389/fpls.2023.1208724 (PMC10413267; doi:10.3389/fpls.2023.1208724)
Supplement: Supplementary file 1 [file Table_1.docx]

Supplementary Material

Effect of micronutrient iron on bioactive compounds isolated form cryptophytes

Maryam Abidizadegan*, Jaanika Blomster, Elina Peltomaa

*** Correspondence:** Maryam Abidizadegan: maryam.abidizadegan@helsinki.fi

# Supplementary Table

**Supplementary Table 1.** Changes in iron content of the algal biomass caused by the treatments were confirmed by iron analysis.

| **Strain** | **Iron level** | **[Fe] % DW** |
| --- | --- | --- |
| *R. salina* | Low | 0.02 |
|  | Medium | 0.03 |
|  | High | 0.04 |
| *C. pyrenoidifera* | Low | 0.01 |
|  | Medium | 0.02 |
|  | High | 0.06 |
| *C. curvata* | Low | 0.01 |
|  | Medium | 0.05 |
|  | High | 0.11 |
| *C. ozolinii* | Low | 0.03 |
|  | Medium | 0.16 |
|  | High | 0.83 |
| *Cryptomonas* sp. | Low | 0.02 |
|  | Medium | 0.03 |
|  | High | 0.24 |

The iron contents of the algal biomass were determined at the end of the experiments from freeze-dried biomass (sample weight 2-70 mg) by mineralizing the samples for 4 hours at 450 °C and dissolving the residue into 10 mL of 5% HCl. The iron analysis was done with atomic absorption spectrometer (AAS; Varian SpectrAA 220 FS, Varian, Palo Alto, CA, USA).
